# Adaptive Gait Training of a Lower Limb Rehabilitation Robot Based on Human–Robot Interaction Force Measurement

**DOI:** 10.34133/cbsystems.0115

**Published:** 2024-06-21

**Authors:** Fuyang Yu, Yu Liu, Zhengxing Wu, Min Tan, Junzhi Yu

**Affiliations:** ^1^ School of Artificial Intelligence, University of Chinese Academy of Sciences, Beijing, China.; ^2^ The Key Laboratory of Cognition and Decision Intelligence for Complex Systems, Institute of Automation, Chinese Academy of Sciences, Beijing, China.; ^3^ School of Mechanical Engineering & Automation, Northeastern University, Shenyang, China.; ^4^ State Key Laboratory for Turbulence and Complex System, Department of Advanced Manufacturing and Robotics, BIC-ESAT, College of Engineering, Peking University, Beijing, China.

## Abstract

The existing fixed gait lower limb rehabilitation robots perform a predetermined walking trajectory for patients, ignoring their residual muscle strength. To enhance patient participation and safety in training, this paper aims to develop a lower limb rehabilitation robot with adaptive gait training capability relying on human–robot interaction force measurement. Firstly, a novel lower limb rehabilitation robot system with several active and passive driven joints is developed, and 2 face-to-face mounted cantilever beam force sensors are employed to measure the human–robot interaction forces. Secondly, a dynamic model of the rehabilitation training robot is constructed to estimate the driven forces of the human lower leg in a completely passive state. Thereafter, based on the theoretical moment from the dynamics and the actual joint interaction force collected by the sensors, an adaptive gait adjustment method is proposed to achieve the goal of adapting to the wearer’s movement intention. Finally, interactive experiments are carried out to validate the effectiveness of the developed rehabilitation training robot system. The proposed rehabilitation training robot system with adaptive gaits offers great potential for future high-quality rehabilitation training, e.g., improving participation and safety.

## Introduction

Due to population aging and various diseases, the number of individuals suffering from lower limb movement disorders gradually increases for the past few years. Traditional rehabilitation training is mainly assisted by medical and nursing professionals, whose effect is limited by the professional abilities and number of support personnel required. Besides, it is challenging to be applied in remote and underdeveloped areas, making it difficult to widely meet the training needs of people with movement disabilities. With the development of robotic technology, rehabilitation training robots have emerged and received increasing attention [1,2]. Many mature products have appeared on the market. For instance, Indego, a wearable lower limb exoskeleton, was developed by Parker Hannifin Corporation for walking rehabilitation training of patients with hemiplegia or spinal damage [3,4]. Ailegs robots, developed by Beijing Da Ai Robotics Co., Ltd., was capable of providing natural gait training to restore muscle movement and balance abilities in patients [5]. Auto LEE was developed as a self-balancing lower limb exoskeleton featuring 10 degrees of freedom [6,7]. Samsung unveiled the GEMS series exoskeleton [8], and Technaid introduced the third-generation robot Exo-H3 [9], boasting more intelligent mobile phone APP control functions. These advancements have effectively expanded the possibilities and accessibility of rehabilitation training for individuals with lower limb movement disorders.

For patients with lower limb movement disorders, their lower limb muscles are weak or unable to actively contract. Rehabilitation training robots can provide patients with auxiliary support and strength, helping to prevent muscle atrophy through repetitive walking and other exercises [10], and ultimately restore partial or complete muscle function. Early rehabilitation robots often use passive training control strategies, such as the Ekso GT exoskeleton robot developed by Ekso Corporation [11], which passively train patients through imitating human walking. Some exoskeleton robots such as Indego [3,4] are equipped with attitude sensors, which can change the passive motion state according to the moving posture. However, passive training methods cannot provide personalized training for patients, and the participation and comfort in recovery training varies from person to person.

In fact, most patients with lower limb movement disorders still retain some residual muscle strength, making complete passive training inefficient as it cannot fully utilize their residual muscle strength [12]. To enhance patient participation in rehabilitation training, exoskeleton robots should be able to sense the patient’s intentions and interact better with them during rehabilitation training. Some researchers have employed electromyographic (EMG) or electroencephalographic (EEG) signals to perceive patients’ motion intentions [13,14]. For instance, Sun et al. [15] utilized surface electromyography to predict knee joint motion intentions and adjust rehabilitation training movements in real time. Li et al. [16] presented an adjustment method for the stair climbing motion of rehabilitation exoskeletons by combining EMG and EEG. Gordleeva et al. [17] proposed a multichannel signal processing method combining EEG and EMG for rehabilitation robots. However, employing EEG and EMG signals requires high acquisition accuracy and stability, leading to complex acquisition equipments. In recent years, some neural-network-based methods have been proposed to process visual information [18,19] and other signals of rehabilitation robots, thus regulating gait phase, joint angle, and joint torque [20–24]. However, these methods often solely focus on predicting the robot’s posture and lack strong perception of the patient’s own motion intentions. Consequently, some studies have investigated human–robot interactions by employing position-based impedance control or force-based admittance control to achieve active motion modes [25,26]. However, in passive rehabilitation training, where the gait training trajectory remains fixed, active patient engagement is limited. To address this issue, it is crucial to dynamically fine-tune the passive gait throughout human–robot interactions. Xu et al. [27] integrated a dynamic movement primitive (DMP) coupled model into mirror therapy, managing the training trajectory of the affected leg via an impedance model, and further refined the coupled DMP model through reinforcement learning to enhance the adaptability to the gait trajectory. Meanwhile, Ma et al. [28] introduced an innovative online gait planning approach leveraging the finite state machine model, which dynamically identifies key gait points through a model based on the transfer of the center of gravity. Further, Xu et al. [29] enhanced the trajectory deformation algorithm and incorporated an auxiliary controller predicated on interaction torque estimation, culminating in improved gait adaptation. More commonly, numerous studies have integrated admittance control strategies within passive training trajectories, facilitating instantaneous gait adjustments. Zhu et al. [30–32] proposed a sliding mode admittance control method to track the position of the human body, enabling the lower limb rehabilitation robot to follow the legs more accurately. Wang et al. [33] applied an impedance control method based on forward dynamics to predict gait and achieved certain results in computer simulation. Unlike electromyography signals, direct measurement of interaction torque is simpler and more reliable. However, during compliance control, there is a tendency to overlook variations among patients and different stages of the gait cycle, resulting in inconsistent utilization of residual muscle force across individuals and movement stages.

Based on the aforementioned observations, this article presents an adaptive gait adjustment method for lower limb rehabilitation robots by introducing human leg dynamics within the framework of the admittance control approach. Combining the active and passive driven styles, a novel lower limb rehabilitation robot system is developed, whose hip and knee joints each have one active degree of freedom, while waist and ankle joints each have one passive degree of freedom. More importantly, 2 face-to-face mounted cantilever beam force sensors are employed to measure the human–robot interaction forces. Thereafter, based on the Lagrange method, a human–robot interaction dynamic model of the developed rehabilitation training robot system is constructed to estimate the driven force of the human leg in a completely passive state. Further, considering the human leg dynamics and residual muscle forces, an adaptive gait regulation method based on admittance control is proposed to realize real-time adaptive gait adjustment of the lower limb rehabilitation robot. Finally, practical interactive experiments are carried out to validate the effectiveness of the developed rehabilitation training robot system as well as the proposed control method, which effectively improves the safety and convenience of rehabilitation training.

The rest of this paper is organized as follows. Materials and Methods describes the mechatronic design of the rehabilitation training robot, proposes a dynamic model of human–robot interaction, and introduces the proposed admittance control method for the training gait. The experiments on human–robot interaction and gait adjustment are conducted in Results and Discussion. Finally, Conclusion summarizes this paper.

## Materials and Methods

### Robot structure and system design

#### Mechatronic design

**Fig. 1.**
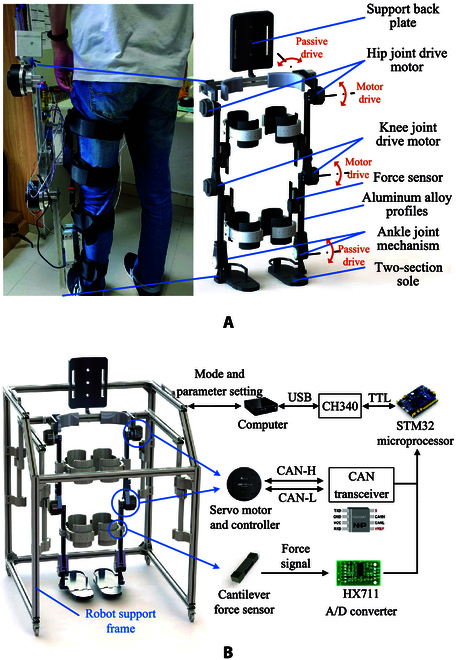
The developed rehabilitation training robot system. (A) Robot structure. (B) Hardware control system.

The developed lower limb rehabilitation training robot is composed of a main robot body and an auxiliary support framework, as shown in Fig. 1. The robot body consists of hip joints, knee joints, ankle joints, connecting rods, and bandages, with a total weight of 22.5 kg. The auxiliary framework is made of aluminium alloy extrusions, which is 1 m in length, 1 m in width, and 1.2 m in height. When used in conjunction with the support frame, the robot is capable of sharing a portion of the user’s weight. In order to connect the patient to the lower limb rehabilitation training robot, the bandages are employed; therefore, the robot can effectively drive the patient’s movement, while the patient’s muscles also generate forces to influence the human–robot interaction. Each side of the robot incorporates 2 passive degrees of freedom located at the waist and ankle, as illustrated in Fig. 1A. The waist degree of freedom ensures unrestricted swinging of the legs in the coronal plane, while the ankle joint degree of freedom permits swinging of the feet in the front and back planes. As shown in Fig. 1A, these 2 active degrees of freedom on each side are powered by motors, with each motor driving one degree of freedom. The hip-joint driven motor is mounted on the lumbar support and securely fastened to the waist to make the thigh swing. The knee-joint driven motor is securely fastened to the thigh, and its output flange converts the motor rotation into lower leg swing. In order to obtain enough continuous torque, the RMD-X8pro motor (Myactuator, Suzhou, China) is employed for these joints, which is rated at 166 W and capable of producing an instantaneous torque up to 25 N·m after deceleration by a 1:9 planetary gear reducer. After deceleration, the peak rotational speed of the motor can reach 160 r/min, which meets the requirements of the rehabilitation training gait cycle of the robot, i.e., 4 to 10 s. Besides, based on the CAN (Controller Area Network) bus communication, the control mode of the employed motors can flexibly select position mode, speed mode, and force control mode on demand. Two passive degrees of freedom on each side are actuated by springs to ensure a safe range. The human ankle joint is a ball and socket joint consisting of the tibia, fibula, and ankle joint surfaces, offering 3 degrees of rotational freedom. Proper positioning of the ankle joint is crucial for ensuring good contact between the foot and the ground during walking [34]. For the purpose of ensuring secure rehabilitation training, the ankle joint of the robot is constrained to rotation within the front and back planes. Additionally, a passive driving method utilizing a spring connection can meet the training requirements. The passive connection cushions the ankle, protects the patient’s ankle, and provides the driven force needed for the foot to lift off and land.

The control system is designed to collect sensor data on interactive forces and adjust the position of the motor in real time. Figure 1B depicts the whole control process. Specifically, the designed controller of the rehabilitation training robot consists of a computer and an STM32F103 microprocessor. Four sets of 8 cantilever force sensors are applied to measure the interactive forces between the rehabilitation training robot and the patient, which is processed by an A/D converter (HX711, a precision 24-bit analog-to-digital converter) and sent to the STM32 microprocessor. After being processed by the control module, the position compensation amount is generated. The microprocessor sends 8-byte real-time position commands to the 4 motors through the CAN bus. The adopted RMD-X8pro is equipped with a motor controller that controls the motor position based on real-time instructions. The computer can modify gait data and control parameters and synchronize real-time parameters to the STM32-based controller every 100 ms.

#### Human–robot interaction force measurement

**Fig. 2.**
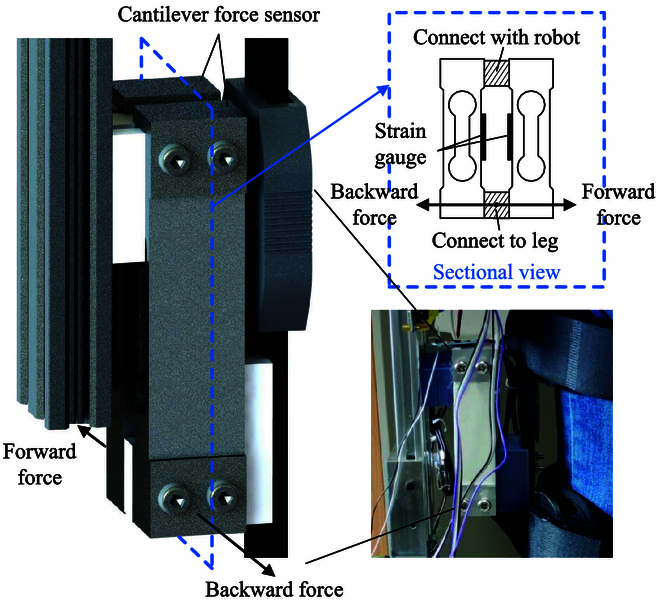
Schematic diagram of the interactive force measurement.

To measure the interaction force between human and the rehabilitation training robot, the developed system incorporates 8 cantilever beam sensors. These sensors are strategically placed in 4 groups on both the left and right legs, responsible for measuring the interaction torque of the hip and knee joints on each side. Notably, the robot features an adjustable height design, and to accommodate this, the sensors are affixed to connecting rods that remain relatively fixed to the measured joints. This design ensures the maintenance of a consistent measuring force arm when adapting to individuals of varying heights. At each measurement position, 2 cantilever force sensors are installed, and 2 strain gauges of every cantilever sensor are installed face-to-face, as illustrated in Fig. 2. The 2 ends of the cantilever beam are separately connected to the robot and leg straps. Thus, the interaction forces between the legs and the robot are easily obtained based on the deformation of the cantilever beam.

It is worth noting that there are 2 sensors installed in reverse to measure the one-way forces acting on the forward and backward directions, respectively. As a result, the interaction force between the robot and the patient causes the deformation in 2 identical cantilever beams; therefore, the actual forces applied should be twice the measurement value of a single sensor. During the sensor installation, a pretension force is applied to ensure the initial value of the sensor is not zero. This approach effectively circumvents the nonlinear behavior observed in small-value readings of cantilever beam sensors. Additionally, each of the 2 sensors installed in a face-to-face arrangement has an individual measurement range of 20 kg. This design allows the sensors to distribute the burden of the interaction force, thus preventing nonlinearity even at high sensor readings. Define *F_forward_* and *F_backward_* as the forward force and backward force of the sensors, respectively. Taking into account the above factors, the actual interaction forces can be obtained as the difference between the 2 measured results on either side. Thereafter, we can obtain the human–robot interaction torque of a single joint as follows:

$$T_{\mathrm{int}}=2\times \left(F_{\text{forward}}-F_{\text{backward}}\right)\times l$$ (1)

where *T_int_* is the human–robot interaction torque; *l* is the distance between the sensor’s leg connection end and the joint.

### Human–robot interaction modeling

In this section, we will conduct the human–robot interaction dynamics of the developed rehabilitation training system, which will provide the solid foundation for the following robot system performance evaluation and the admittance controller construction.

**Fig. 3.**
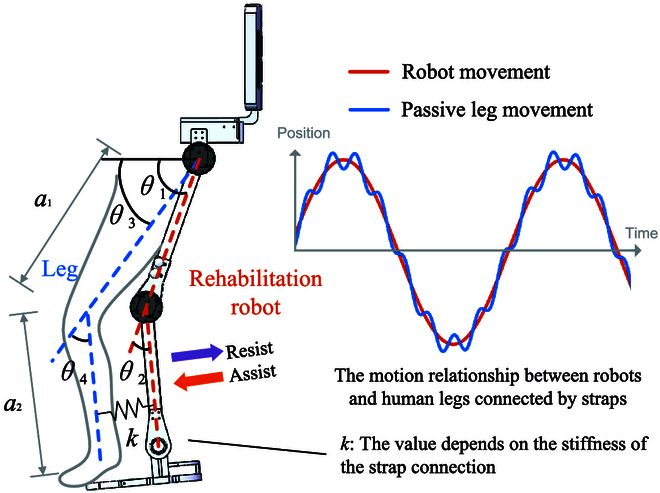
Human–robot coupling model.

Figure 3 illustrates the human–robot coupling model. In particular, *θ*_1_ and *θ*_2_ denote the hip and knee joint angles of the robot, respectively, while *θ*_3_ and *θ*_4_ represent the corresponding angles for the human leg. Define Θ = [*θ*_1_, *θ*_2_]^T^ to represent the joint coordinate vector of the robot. *a*_1_ and *a*_2_ denote the length of the thigh and calf links, respectively. Irrespective of the human leg’s involvement, the exoskeleton is considered as an independent system, and its torque output is generated by joint motors. Define *T_r_* = [*τ*_1_, *τ*_2_]^T^ to represent the torque output vector of the robot. The weights of the robot’s thigh and calf links are denoted as *m*_1_ and *m*_2_, respectively. We assume that the center of mass for each link is located at the center of the link itself. As a result, the centroids of the 2 links are positioned at a distance of *a*_1_/2 and *a*_2_/2, respectively.

The dynamic model of the lower limb rehabilitation training robot is constructed based on Lagrange equations. The Lagrange function is defined as the difference between the kinetic energy and potential energy of the system, expressed as *L* = *K* − *P*, where *K* and *P* represent the system’s kinetic and potential energies, respectively. Based on the Lagrange method, it is easy to obtain the dynamic model of the lower limb rehabilitation training robot as follows:

$$\begin{array}{c} \begin{array}{ll} \left[\begin{array}{c} \tau_{1}\\ \tau_{2} \end{array}\right] & =T_{\mathrm{dis}}+f\left(\overset{\cdot }{\Theta }\right)+M\left(\Theta \right)\left[\begin{array}{c} {\ddot{\theta }}_{1}\\ {\ddot{\theta }}_{2} \end{array}\right]\\ & +\frac{1}{2}m_{2}a_{1}a_{2}\text{sin}\theta_{2}\left[\begin{array}{c} -2{\overset{\cdot }{\theta }}_{1}{\overset{\cdot }{\theta }}_{2}-{\overset{\cdot }{\theta }}_{2}^{2}\\ {\overset{\cdot }{\theta }}_{1}^{2} \end{array}\right]+G\left(\Theta \right) \end{array} \end{array}$$ (2)

where *T_dis_* represents external disturbances to the robot system, $f\left(\overset{\cdot }{\Theta }\right)$ represents system friction, and it is related to joint motion speed. *M*(Θ) is the inertia matrix of the robot, and *G*(Θ) is the gravity matrix, expressed as follows:

$$\begin{array}{c} \begin{array}{cc} M\left(\Theta \right) & =\left[\begin{array}{cc} M_{11} & M_{12}\\ M_{21} & M_{22} \end{array}\right]\\ G\left(\Theta \right) & =\left[\begin{array}{c} G_{11}\\ G_{21} \end{array}\right]\\ M_{11} & =\left(\frac{1}{4}m_{1}+m_{2}\right)a_{1}^{2}+\frac{1}{4}m_{2}a_{2}^{2}+m_{2}a_{1}a_{2}\text{cos}\theta_{2}\\ M_{12} & =\frac{1}{4}m_{2}a_{2}^{2}+\frac{1}{2}m_{2}a_{1}a_{2}\text{cos}\theta_{2}\\ M_{21} & =\frac{1}{4}m_{2}a_{2}^{2}+\frac{1}{2}m_{2}a_{1}a_{2}\text{cos}\theta_{2}\\ M_{22} & =\frac{1}{4}m_{2}a_{2}^{2}\\ G_{11} & =\left(\frac{1}{2}m_{1}+m_{2}\right)ga_{1}\text{cos}\theta_{1}\\ & \;+\frac{1}{2}m_{2}ga_{2}\text{cos}\left(\theta_{1}+\theta_{2}\right)\\ G_{21} & =\frac{1}{2}m_{2}ga_{2}\text{cos}\left(\theta_{1}+\theta_{2}\right) \end{array} \end{array}$$ (3)

In the robot system, in addition to the influence of gravity, there is also interaction force between the robot and human legs. To account for this, a human–robot coupling model is established based on the theoretical interaction force estimation method [30]. In the coupling model, the motors serve as the power source, and the human legs do not generate active muscle force. As a result, the Lagrange equation incorporating passive leg participation can be expressed as follows:

$$\begin{array}{c} \begin{array}{ll} T_{r} & =T_{\mathrm{dis}}+f\left(\overset{\cdot }{\Theta }\right)+T_{h}+M\left(\Theta \right)\left[\begin{array}{c} {\ddot{\theta }}_{1}\\ {\ddot{\theta }}_{2} \end{array}\right]\\ & +\frac{1}{2}m_{2}a_{1}a_{2}\text{sin}\theta_{2}\left[\begin{array}{c} -2{\overset{\cdot }{\theta }}_{1}{\overset{\cdot }{\theta }}_{2}-{\overset{\cdot }{\theta }}_{2}^{2}\\ {\overset{\cdot }{\theta }}_{1}^{2} \end{array}\right]+G\left(\Theta \right) \end{array} \end{array}$$ (4)

where *T_h_* represents the interactive torque of the human leg to the robot system when no muscle force is exerted. Based on the stiffness modeling method for human–robot connections presented in [35,36], we suppose the existence of a fixed stiffness connection between the human leg and the robot. The corresponding motion relationship is illustrated in Fig. 3. The interaction force between the human leg and the robot is established based on an elastic relationship, and the magnitude of this interaction force is intricately linked to the stiffness and displacement difference of the human–robot connection. Since the angular difference between the human and the robot is small, the torque magnitude can be expressed in terms of the stiffness coefficient and deformation.

$$\begin{array}{c} \begin{array}{l} T_{h}=\left[\begin{array}{c} T_{h1}\\ T_{h2} \end{array}\right]\\ T_{h1}=k{\left(\frac{a_{1}}{2}\right)}^{2}\text{sin}\left(\theta_{1}-\theta_{3}\right)+k\left[a_{1}\text{sin}\left(\theta_{1}-\theta_{3}\right)\right.\\ +\frac{a_{2}}{2}\text{sin}\left.\left(\theta_{2}-\theta_{4}\right)\right]\left(a_{1}\text{cos}\;\theta_{2}+\frac{a_{2}}{2}\right)\\ T_{h2}=k\left[a_{1}\text{sin}\left(\theta_{1}-\theta_{3}\right)+\frac{a_{2}}{2}\text{sin}\left(\theta_{2}-\theta_{4}\right)\right]\frac{a_{2}}{2} \end{array} \end{array}$$ (5)

where *k* is the elastic coefficient between the lower limb of the human body and the robot, determined by the connection method between the robot and the leg. Obviously, its numerical value represents the system stiffness.

In practice, it is difficult to calculate the driven torque of the leg generated by elasticity through either an accurate model or direct measurement. Considering that the human leg is entirely propelled by the robot to generate motion in passive training mode, we simplify the interaction force generated by the strap connection as the force driving the movement of the human leg. To address this, we have developed a dynamic model to estimate the driving torque of the legs in the absence of muscle forces, with the legs serving as the primary focus of the model.

$$\begin{array}{c} \begin{array}{cc} T_{h} & =M_{h}\left[\begin{array}{c} {\ddot{\theta }}_{3}\\ {\ddot{\theta }}_{4} \end{array}\right]\\ & +\frac{1}{2}m_{l}a_{1}a_{2}\text{sin}\theta_{4}\left[\begin{array}{c} -2{\overset{\cdot }{\theta }}_{3}{\overset{\cdot }{\theta }}_{4}-{\overset{\cdot }{\theta }}_{4}^{2}\\ {\overset{\cdot }{\theta }}_{3}^{2} \end{array}\right]+G_{h} \end{array} \end{array}$$ (6)

where *M_h_* is the inertia matrix of human lower leg, *G_h_* is the gravity matrix, and *m_l_* represents the theoretical weight of the lower leg.

### Adaptive gait training method based on admittance control

In the human–robot coupled dynamic model, the joint motors provide driven torques, and the leg is in a state of no muscle force. However, during actual rehabilitation trains, it should consider the participation of residual muscle strength in the human leg. Training without residual muscle strength is entirely passive. In a passive training state, when a muscle generates force in the same direction as the leg moves, the robot will restrict the range of motion of the patient; otherwise, it will impede the muscle’s ability to exert forces. To address this issue, an impedance model is introduced to describe the relationship between the rehabilitation training robot and the human leg, which utilizes the forces generated by the human leg for compliance control of the robot.

$$M_{d}\Delta \ddot{\Theta }+B_{d}\Delta \overset{\cdot }{\Theta }+K_{d}\Delta \Theta =T_{\mathrm{ad}}$$ (7)

where ΔΘ represents the difference between the current joint angle and the target angle. *M_d_*, *B_d_*, and *K_d_* are preset admittance parameters that separately represent the rotational inertia, damping, and stiffness matrices of the rehabilitation training robot interacting with the human leg. *T_ad_* = *T_int_* − *T_h_* represents the magnitude of residual muscle force of the leg, which is the difference between the actual human–robot interaction torque collected by the sensors and the theoretical torque of the dynamic model. When human muscles exert force, there is a difference between the actual interaction force and the theoretical value. To comply with the patient’s movement trend, the joint position and speed can be solved and adjusted in real time based on Eq. 7. Appearently, the admittance characteristics of the rehabilitation training robot represent its ability to track the human leg’s movements.

According to Eq. 7, it is evident that in the admittance control model, the input is the interaction force *T_int_*, and the output is the position error Δ*q*. The robot system exhibits second-order characteristics and can be expressed as:

$$\begin{array}{c} \begin{array}{ll} & s^{2}+2\xi \omega_{n}s+\omega_{n}^{2}=0\\ & \;\xi =\frac{B_{d}}{2\sqrt{M_{d}K_{d}}}\\ & \;\omega_{n}=\sqrt{\frac{K_{d}}{M_{d}}} \end{array} \end{array}$$ (8)

where *ξ* is the damping ratio and *ω_n_* is the natural frequency of the system. The stability of the control model can be ensured by modifying the admittance parameters [37].

**Fig. 4.**
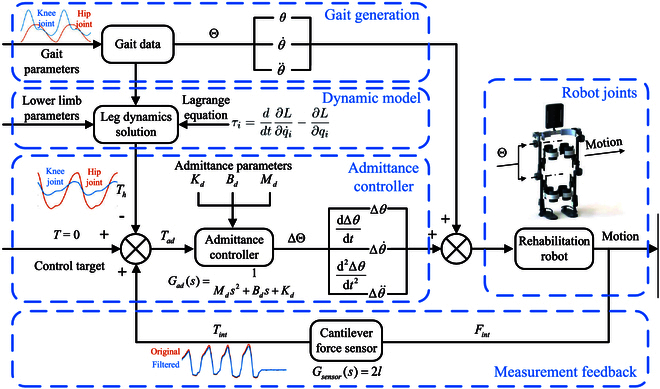
Framework of the adaptive gait control system.

Figure 4 shows the whole adaptive gait training process based on admittance control for the developed rehabilitation training robot. In gait generation state, according to the collected gait data, the joint angle state Θ can easily be obtained. Thereafter, based on the constructed kinematic and dynamic models of the human leg, the human–robot interaction torque *T_h_* without any muscle force is calculated. Subsequently, the admittance controller starts to work. Specifically, the input control target is set as *T* = 0. It means the difference between the human–robot interaction torque *T_h_* and *T_int_* is fully utilized as admittance control to minimize the obstruction caused by the robot to the human leg movement. Meanwhile, the admittance control module calculates the real-time angle adjustment amount ΔΘ of the joints. These calculated results are superposed with the theoretical joint angles to obtain a new real-time gait. Under actions of the employed servo motors, these newly generated gaits can be easily and effectively executed by the rehabilitation training robot. During the whole time, the installed cantilever force sensors will collect the interactive forces and calculate *T_int_* for feedback.

## Results and Discussion

### Lower limb gait collection

As for the lower limb rehabilitation training robots, no matter what control strategy employed, human gait curves are very important. Only having suitable gait curves, the rehabilitation training robots can aid the patients better in their recovery. In actual operation, more important for gait curves lies in their generation and testing for stability [38]. Many factors will affect the extraction of the gait curves, such as the accuracy and stability of the sensors and environmental interference. Therefore, it is necessary to filter the extracted gait data to ensure the stability of the gait curves. Following the existing gait curves generation approach [39], we collected 9 key positions of the hip joint and 12 key positions of the knee joint within a gait cycle of each joint. Table tabulates the collected serialized positions of both knee joint and hip joint. These key points are more densely located at positions with large curve change rates, and the redundancy of key points ensures the stability and smoothness of the generated gait.

**Table.** Serialized Positions of the knee and hip joint in a gait cycle

Knee joint		Hip joint	
Cycle proportion	Angle (°)	Cycle proportion	Angle (°)
0	8.18347	0	−15.79609
0.11	34.53819	0.04	−14.00131
0.22	58.68233	0.21	11.52780
0.24	58.78970	0.41	24.87653
0.41	22.20942	0.43	24.87214
0.43	18.53719	0.5	24.72710
0.5	13.34561	0.89	−10.29988
0.57	14.36569	0.9	−10.97715
0.59	14.32100	1	−15.79609
0.89	0		
0.9	0.30874		
1	8.18347		

**Fig. 5.**
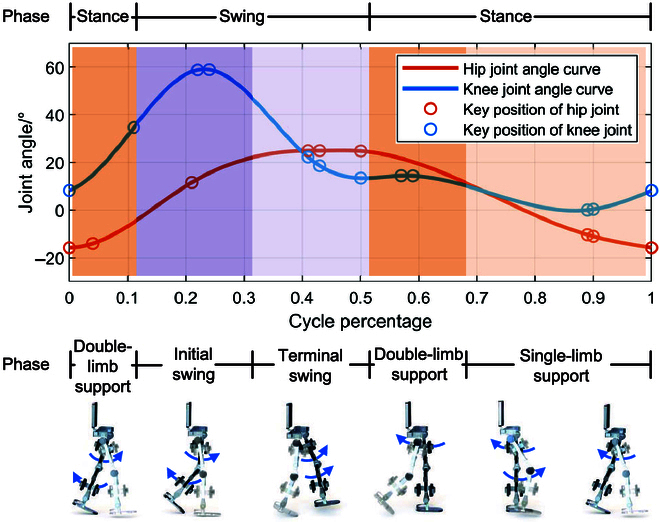
The key positions and interpolation results of knee and hip joints.

Through performing the cubic spline interpolation of these collected joint angles, i.e., 9 positions of the hip joint and 12 positions of the knee joint, we can obtain the final gait curves, as shown in Fig. 5. Taking the left leg for example, we can easily observe 2 obvious stages from the gait curves, i.e., stance and swing, which is visually depicted in orange and purple background colors in Fig. 5. As for the stance stage, it can be further divided into a single-leg stance phase and a double-leg stance phase. As the right leg steps forward, the left leg transitions into a single-leg stance. Subsequently, it shifts to a double-leg stance along with the right leg lands. As for the swing stage, it is composed of an initial phase and a final phase. The former commences as the left foot lifts off, and the latter starts as the center of gravity readjusts. Figure 5 illustrates the motion sequence diagram of the whole process, demonstrating relatively smooth motion simulation results, thus serving as the fundamental gait for rehabilitation training experiments.

### Human–robot interaction experiment

The following experiments focus on the human–robot interaction force measurement and analysis based on the admittance control method. According to the interaction information between the patient’s legs and the rehabilitation training robot, the admittance control is employed to regulate the positions of the knee and hip joints of the robot, thus completing the force-position control process [40]. The relationship between the interaction force and joint angles is easily established based on Eq. 7. Meanwhile, using the installed cantilever force sensors, we can directly measure the interaction forces between the lower leg and the robot. Here, we take the left knee joint as the experimental object. To test the effect of the employed admittance control method, we fixed the gait curve of the knee joint, and set the input gait angle to always be 0°, that is, Θ = 0. The servo motor of knee joint was operated in position control mode, receiving position commands via the CAN bus. In the calculation of the admittance model, we processed the first- and second-order differentials in the form of positional differences, with a period of 100 ms.

**Fig. 6.**
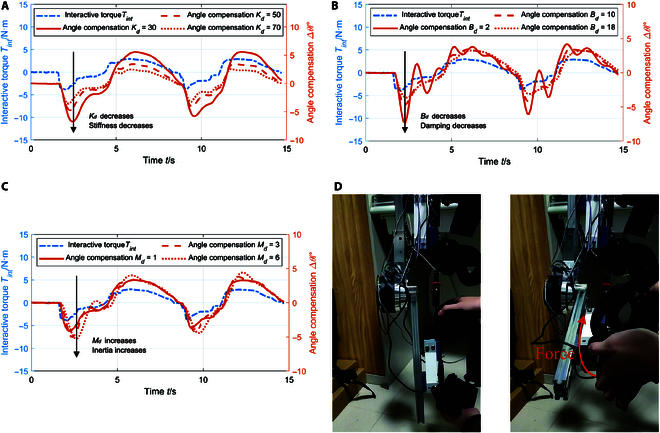
The results of human–robot interaction experiments. (A) Results of changing *K_d_* with a fixed *B_d_* = 10 N · m · s/rad and *M_d_* = 2 N · m · s^2^/rad. (B) Results of changing *B_d_* with a fixed *K_d_* = 50 N · m/rad and *M_d_* = 2 N · m · s^2^/rad. (C) Results of changing *M_d_* with a fixed *K_d_* = 50 N · m/rad and *B_d_* = 10 N · m · s/rad. (D) Guiding force is applied to the robot system.

According to Eq. 8, we can easily make out that in admittance model, the system performance is decided by some key parameters, for example, *K_d_* for stiffness, *B_d_* for damping, and *M_d_* for inertia. Through changing these key system parameters, we can obtain different angle compensation response characteristics. Therefore, it is very important to explore how these parameters affect the system performance. Figure 6 illustrates the experimental results of the built robotic system in varying key parameters. More concretely, the first tests were executed under the conditions of changing *K_d_* with a fixed *B_d_* = 10 N · m · s/rad and *M_d_* = 2 N · m · s^2^/rad, as shown in Fig. 6A. We can find that as *K_d_* increases, the angle compensation amount obviously decreases. Therefore, it is crucial to increase *K_d_* to limit angle compensation range appropriately to ensure system stability. Figure 6B depicts the outcomes of changing *B_d_* with a fixed *K_d_* = 50 N · m/rad and *M_d_* = 2 N · m · s^2^/rad. We can find that as *B_d_* increases, the system response slows down, but the fluctuation decreases. Therefore, increasing *B_d_* appropriately prevents overshoot of the joint when it reaches its extreme point, thus improving training safety. With a fixed *K_d_* = 50 N · m/rad and *B_d_* = 10 N · m · s/rad, the system inertia increases and the response slows down as *M_d_* increases, as shown in Fig. 6C. In active mode admittance control, tracking error is commonly employed as an assessment indicator for parameter selection [41]. In our study, we employed admittance models and human dynamic models to implement trajectory-adaptive control in passive training. Consequently, parameter selection is conducted to adjust the passive training trajectory mode based on interaction force, aiming to maximize patient engagement while simultaneously ensuring safety. Based on the experimental results, the parameters of the adaptive rehabilitation gait control system are set to *K_d_* = 50 N · m/rad, *B_d_* = 10 N · m · s/rad, and *M_d_* = 2 N · m · s^2^/rad.

### Gait optimized by admittance method

In the following experiments, a volunteer is employed to wear the developed rehabilitation training robot to do some tests. The volunteer is 180 cm tall and weighs 60 kg. According to the human body parameter estimation method in [42], we can estimate the dynamic parameters of the volunteer, taking the thigh mass as 8.5 kg, the calf mass as 4.5 kg, the thigh length as 0.37 m, and the calf length as 0.47 m. Meanwhile, assume uniform mass distribution along the human leg.

**Fig. 7.**
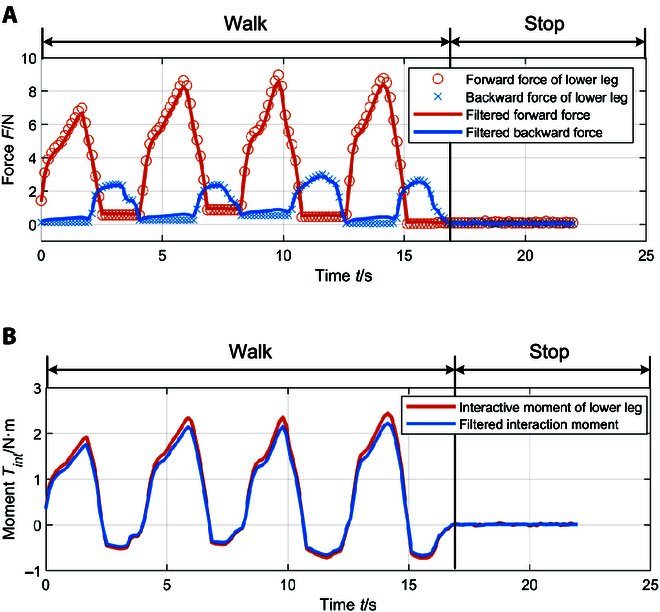
Measured knee joint interaction forces and moments. (A) Lower leg force sensor data and filtered results. (B) Human–robot interaction moment of knee joint.

At first, the interactive forces in both direction of the left knee joint during walking training can be collected by the installed cantilever force sensors. In the test, the training gait cycle was set to 4 s. Figure 7A displays the cantilever force sensor data collected within 4 walking cycles. Due to installation errors, connection stiffness, and other factors, the obtained sensor data exhibited poor stability. To compensate for errors caused by sensor measurements, Kalman filtering approach was employed to perform recursive data processing, contributing to enhanced signal stability and reduced sensor errors [43]. After processing the data, the human–robot interaction moments of the left knee joint during 4 gait cycles were obtained, as shown in Fig. 7B. In particular, the interaction moments are depicted by the blue and red curves, which represent the moment changes before and after filtering, respectively.

**Fig. 8.**
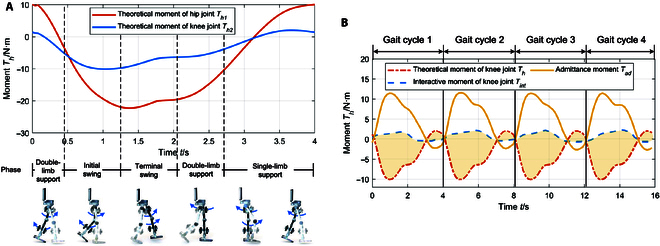
The curves of theoretical moment and admittance moment. (A) Theoretical moment *T_h_* of joint in 1 cycle. (B) Admittance moment in 4 cycles.

To derive the admittance equations, it is necessary to calculate the driving forces of the human leg in the absence of any muscle force. To achieve this, a dynamic model of the human leg is established based on Eq. 6. Through solving the theoretical interaction moment *T_h_* throughout a gait cycle (4 s), we can obtain the 2 joint moments displayed in Fig. 8A. These results are consistent throughout the cycle and similar to the walking moment estimation outcomes for normal individuals in [42], demonstrating the feasibility of the constructed estimation method in Eq. 6. The relationship between the left knee joint interaction force and admittance control force was analyzed under experimental conditions. According to *T_ad_* = *T_int_* − *T_h_*, we can obtain the changes in *T_ad_* of the left knee joint over 4 cycles, as shown in Fig. 8B. We know that *T_ad_* is a system moment in knee joint admittance control. Since the volunteer is a healthy individual during the experiment, there are typically substantial active muscle force during walking; therefore, *T_int_* exerted on the rehabilitation training robot was mostly opposite to the theoretical moment *T_h_* when the patient has no muscle force. In such a scenario, the admittance moment *T_ad_* represented greater compensation to the human leg in the same direction as the motion. According to Eq. 7, we performed a differential calculation to calculate the compensation angle ΔΘ. Considering the results of the human–robot interaction experiment above, we set the parameters as follows: *K_d_* = 50 N · m/rad, *B_d_* = 10 N · m · s/rad and *M_d_* = 2 N · m · s^2^/rad.

**Fig. 9.**
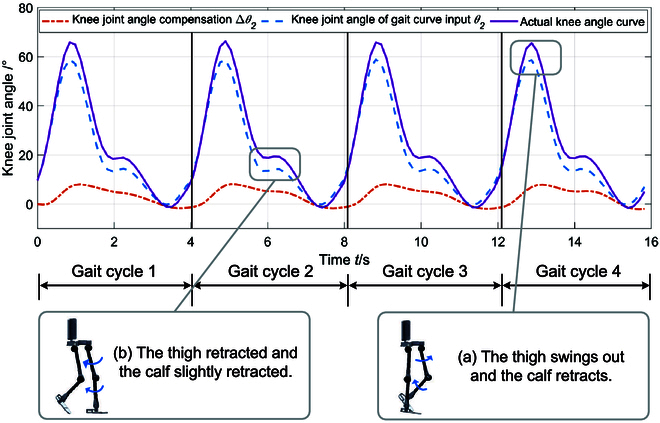
Compensation results of knee joint angle.

Figure 9 presents the input curve and compensation results of the left knee joint during the experiment. As the volunteer was healthy, the leg muscle strength caused the forward and backward movements to occur ahead of the input curve. However, the admittance control system was able to predict the leads and adjust the gait curve through angle compensation. As a result, the range of joint motion in the real-time adjusted curve was larger, which met the expectations for adjustment.

### Discussion

The proposed admittance-control-based adaptive gait training method, which takes into account human leg dynamics, proves to be highly beneficial in harnessing the residual muscle strength of the disable lower limb. Through optimizing the training gait in real time, this developed rehabilitation training robot effectively enhances patient participation and safety, while ensuring an effective training. In comparison to fully active control methods like those in [44], adaptive passive training methods offer more stable training curves, ensuring both safety and stability. While existing passive rehabilitation training methods [45,46] can achieve gait adjustment during training, they often struggle to fully utilize residual muscle strength. By incorporating human leg dynamics, the proposed training process can adapt to diverse individuals and stages, thereby enhancing training participation. In this study, we conducted interactive experiments using a human–robot dynamic model and estimated optimal admittance parameters. The subsequent rehabilitation training experiments, encompassing 4 gait cycles, showed the stationarity and efficacy of the proposed adaptive gait method.

However, the reliance on a basic gait pattern and the current 10-Hz control frequency limit our ability to accurately access and respond to the patient’s other motion intentions during rehabilitation training, such as initiating and halting walking movements. In order to address this issue, more diverse ranges of training postures should be incorporated into the basic gait pattern. Furthermore, the patient’s motion frequency is set as a preset value, necessitating further exploration in enhancing the adaptability of the admittance control parameters to accommodate individual variations in walking frequencies. By enhancing the gait adaptation ability of the rehabilitation training robot, we can better tailor the training program to meet the unique needs of each patient.

## Conclusion

In this paper, we have developed a novel lower limb rehabilitation training robot with the abilities of adaptive gait adjustment. In order to obtain the joint interaction torque, the developed lower limb rehabilitation training robot employs 2 face-to-face mounted cantilever beam force sensors to measure the human–robot interaction forces in 2 directions. Relying on Lagrange equations, a dynamic model of the human–robot interaction system is constructed to estimate the driven force of the human leg in a completely passive state. Thus, the residual muscle force of the human leg can be obtained through comparing the theoretical moment with the actual joint interaction force collected by the sensors, and the real-time joint position compensation angle can be obtained after processing the admittance moment generated by the residual muscle force. Based on this, an adaptive gait adjustment method is proposed to achieve the goal of adapting to the wearer’s movement intention. Several experiments are carried out to verify the developed lower limb rehabilitation training robots. The results shows that the adaptive gait training method based on admittance control can effectively improve the rehabilitation training engagement and safety.

Future work will focus on analyzing the disparities in gait cycles between rehabilitation robots and patients, fine-tuning the robot’s gait speed to better accommodate the patient’s walking pace, and enhancing the safety and engagement of rehabilitation training.

## Data Availability

The data used to support the findings of this study are available from the corresponding author upon request.
